# Heterogeneity Confounds Establishment of “a” Model Microbial Strain

**DOI:** 10.1128/mBio.00135-17

**Published:** 2017-02-21

**Authors:** Nancy P. Keller

**Affiliations:** Department of Medical Microbiology and Immunology, University of Wisconsin—Madison, Madison, Wisconsin, USA

## Abstract

*Aspergillus fumigatus* is a ubiquitous environmental mold and the leading cause of diverse human diseases ranging from allergenic bronchopulmonary aspergillosis (ABPA) to invasive pulmonary aspergillosis (IPA). Experimental investigations of the biology and virulence of this opportunistic pathogen have historically used a few type strains; however, it is increasingly observed with this fungus that heterogeneity among isolates potentially confounds the use of these reference isolates. Illustrating this point, Kowalski et al. (mBio 7:e01515-16, 2016, https://doi.org/10.1128/mBio.01515-16) demonstrated that variation in 16 environmental and clinical isolates of *A. fumigatus* correlated virulence with fitness in low oxygen, whereas Fuller et al. (mBio 7:e01517-16, 2016, https://doi.org/10.1128/mBio.01517-16) showed wide variation in light responses at a physiological and protein functionality level in 15 *A. fumigatus* isolates. In both studies, two commonly used type strains, Af293 and CEA10, displayed significant differences in physiological responses to abiotic stimuli and virulence in a murine model of IPA.

## COMMENTARY

The genus *Aspergillus*, first described in 1729 by the Italian priest and biologist Pier Antonio Micheli, consists of well over 300 species (1) and has a long and impactful history of interaction with humans. Well-known members of this genus include the genetic model *A. nidulans*, the industrial workhorses *A. niger* and *A. terreus*, the mycotoxigenic plant pathogens *A. flavus*, *A. parasiticus*, and *A. ochraceus*, the food fermenters *A. oryzae* and *A. sojae*, and the opportunistic human pathogen *A. fumigatus*. *Aspergillus fumigatus* was coined as a distinct species by physician J. B. Georg W. Fresenius in 1863 (2) and is recognized as the most common *Aspergillus* species to infect humans. This saprophyte is a successful colonizer of decaying organic matter in soils and detritus worldwide, producing an untold number of infectious spores in nearly all milieus accessible to human habitats, including even the international space station (3).

Clinical presentations of *Aspergillus* infection vary widely and often correlate with states of altered patient immunity. For example, allergenic bronchopulmonary aspergillosis (ABPA) represents a heightened immune response to *Aspergillus* spores and most commonly affects asthma, cystic fibrosis, and bronchiectasis patients (4). Chronic pulmonary aspergillosis (CPA) occurs in otherwise immunologically healthy patients and is frequently associated with the development of “fungal balls” known as aspergillomas, which can develop in preexisting cavities caused by other pulmonary ills (http://www.aspergillus.org.uk/content/aspergilloma-0). The most severe disease, invasive pulmonary aspergillosis (IPA), is characterized by abundant fungal growth beyond the lung tissues and occurs most often in immunocompromised patients. The worldwide frequency and profiles of patients at risk for IPA continues to expand, owing to increased numbers of immunocompromised patients, including those afflicted with HIV/AIDS, malignancy, leukemia, organ dysfunction, and immunosuppressive medical treatments. Antifungal treatment is the only option for IPA patients, but fatality rates remain between 30 and 90% (5). Coupled with these grim statistics is the concern over an increasing number of antifungal-resistant isolates (6).

Although *A. fumigatus* has long been recognized as the leading cause of IPA, understanding the pathogenesis of this difficult-to-treat pathogen remained largely unknown until the advent of its genome sequence (the first *A. fumigatus* strain was sequenced in 2005 [7]), coupled with the development of enhanced molecular tools allowing for ready deletion, mutation, or overexpression of *A. fumigatus* genes (8, 9). Most advances have focused on two commonly researched isolates of *A. fumigatus*: Af293 and CEA10 (or their derivatives) (http://aspergillusgenome.org/Strains.shtml). An early comparison (2007) of the genomes of these two strains showed that while most regions of the two genomes displayed high synteny, Af293 contained 208 and CEA10 (also called A1163) contained 320 unique genes not found in the assembled genome of the other strain (10). At the time, this analysis did not explore more subtle differences, such as single-nucleotide polymorphisms (SNPs) or indels. Cruder techniques had foreshadowed these findings (11, 12), and subsequent studies of genomes of diverse *A. fumigatus* strains delved into SNP and indel analysis of specific loci, such as *cyp51A*, associated with azole resistance (13). In addition, a comprehensive comparison of deletions, insertions, multiple-nucleotide variations, replacements, and single-nucleotide variations within 26 toxin gene clusters of two space station isolates, as well as of Af293 and CEA10, was done (3). This comprehensive analysis identified 1,578 variations from Af293’s sequence in the two space station isolates and CEA10 and corroborated previously published mutations that result in the loss of fumitremorgin production in Af293 (14) and trypacidin production in CEA10 (15), and it identified unique mutations in the space station isolates likely affecting toxin production.

These findings, coupled with the many reports of significant physiological differences in the levels of growth and virulence of *A. fumigatus* isolates support a view that pathogen heterogeneity may underlie some of the disease variability reported in the literature (16–18), including that noted by an initial study demonstrating differential immunogenic responses to multiple *A. fumigatus* strains (including Af293 and CEA10) (19). However, some studies have attributed experimentally observed differences in pathogenicity to the host model and even professed that *A. fumigatus* infections may be a product solely of host status and immune response (20). There is no doubt that host immune status is critical in *A. fumigatus* pathogenesis, as illustrated in studies using diverse animal models of invasive aspergillosis, such as *Drosophila melanogaster* (21), *Galleria mellonella* (18), zebrafish (22), and mice (17). In zebrafish and murine models, the methods used to generate immunosuppressed hosts demonstrate a clear effect on pathogenic manifestation. A key example of the influence of host immunity on disease outcome centers around five different studies assessing the virulence of independent gliotoxin mutants, where four *A. fumigatus* strains (including Af293 and the CEA10 derivative CEA17) and two murine regimes (neutropenic and nonneutropenic) were assessed (reviewed in reference 23). Regardless of fungal isolate, the gliotoxin mutants were less virulent only in the nonneutropenic models, thus increasing confidence in a gliotoxin-neutrophil interaction in IPA’s outcome.

However, most studies remain cloudy as to the relative contributions of the host and the *A. fumigatus* strain. Kowalski et al. (24) tackle this conundrum head-on in addressing the hypothesis that heterogeneity in* A. fumigatus* growth in low-oxygen environments underlies virulence, dependent on host immune status. Building on prior data supporting *A. fumigatus*’s ability to grow in hypoxic environments as a virulence determinant (reviewed in reference 25), this study rigorously confirmed and expanded these earlier findings by using multiple clinical and environmental strains of *A. fumigatus*. The researchers demonstrate an enhanced hypoxic survival ability of CEA10 over Af293, illustrated by a higher fitness ratio of biomass in hypoxia to biomass in normoxia (H/N ratio) in CEA10. As their earlier research had also demonstrated increased hypoxic regions in lungs of nonneutropenic mice versus those of neutropenic mice (26), the authors speculated that CEA10 might show increased virulence over Af293 in a nonneutropenic murine model but not in a neutropenic model. This was borne out in virulence studies. They next obtained an additional 14 strains of *A. fumigatus* and were able to correlate a higher H/N ratio with increased virulence in the nonneutropenic model. This work was elegantly topped off with an evolution test where Af293 was passaged for 20 generations in increasing-hypoxia environments to generate EVOL20, an evolved strain with a higher H/N fitness ratio than its parent’s, Af293, and virulence attributes more similar to those of CEA10 and other high-H/N strains. In addition to firmly linking low-oxygen attributes to virulence, this study provides strong evidence that environmental pressures on a heterogenous population select *A. fumigatus* traits that can have subsequent impacts on virulence.

Complementing the Kowalski et al. study, Fuller et al. analyzed the light responses in 15 *A. fumigatus* isolates, again including the standards Af293 and CEA10 (27). Many developmental programs are regulated by light in fungi and in the genus *Aspergillus*; light-mediated pathways have been well established in the genetic model *A. nidulans.* In this model species, light promotes asexual reproduction by inhibiting the activity of a blue-light photoreceptor (LreA) while promoting the activity of the red-light photoreceptor (FphA). Initial studies by this group indicated that this light-driven asexual sporulation process was not conserved in *A. fumigatus* despite the presence of both LreA and FphA orthologs in the genome (28). Instead, LreA drove the synthesis of mycelial pigmentation, and FphA was required for germination and a cell wall homeostatic response to light. These responses originally led the authors to conclude that light served as a stress-signaling mechanism in *A. fumigatus* rather than having a developmental role, as in *A. nidulans*. However, all of this work was derived from studies of Af293. Considering the reported variation in different *A. fumigatus* strains, Fuller et al. expanded their scope to delve into light response using 15* A. fumigatus* isolates. These 15 isolates fell largely into two light response groups: one where light drove hyperpigmentation, as previously described for Af293, and a second one, which included isolate CEA10, where asexual sporulation was induced by light, similar to the behavior in *A. nidulans*. Interestingly, LreA was key for both behaviors, thus illustrating a differential role for this blue-light receptor in distinct isolates of *A. fumigatus*. Unlike in the Kowalski et al. study, heterogeneity in the light responses did not track with virulence in the nonneutropenic model, nor was it associated with the presence of LreA.

**FIG 1:**
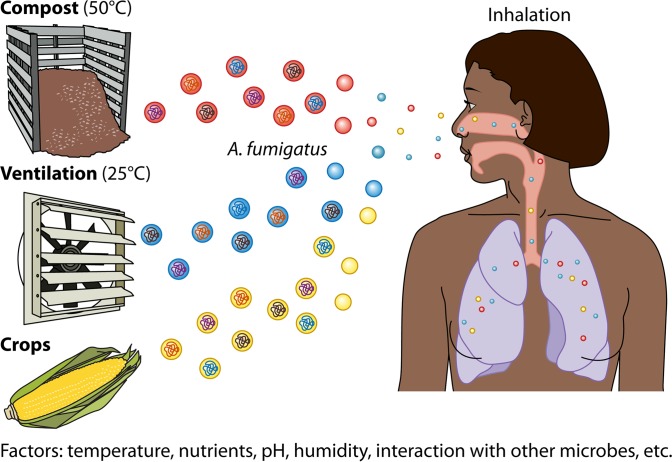
*Aspergillus fumigatus* thrives in numerous environmental niches. Spores from diverse environments are ubiquitous in the air that we breathe. Each *A. fumigatus* population adapts to unique habitats in response to different abiotic and biotic stresses. These adaptations can drive heterogeneity in the fungus and fungal isolates.

Both studies serve to demonstrate that care must be taken in drawing conclusions on organismal developmental processes or symbioses (parasitic or otherwise) from use of a single genetic background. This point may be particularly true for opportunistic pathogens like *A. fumigatus*, which flourish in a variety of environments, each with its own unique microbiome. The heterogeneity of any *A. fumigatus* isolate likely reflects adaptations to the biotic and abiotic pressures of that particular environment (Fig. 1). The most advantageous traits for surviving in a particular niche, e.g., temperature tolerance, toxin production, UV resistance, enzymatic repertoire, hypoxia, etc., differ and may significantly impact the virulence of *A. fumigatus* strains originating from such environments. This raises the question of whether pathogens adapted to and largely found only on humans (e.g., *Candida* spp.), in contrast to such environmentally adaptable pathogens as *A. fumigatus*, exhibit less heterogeneity and, consequently, less plasticity in virulence. Regardless of this possibility, certainly a dialogue on this conundrum of which and how many *A. fumigatus* strains should be assessed in research efforts is ripe for discussion.
